# Global fitting of multiple data frames from SEC–SAXS to investigate the structure of next-generation nanodiscs

**DOI:** 10.1107/S2059798322001838

**Published:** 2022-03-11

**Authors:** Abigail Barclay, Nicolai Tidemand Johansen, Frederik Grønbæk Tidemand, Lise Arleth, Martin Cramer Pedersen

**Affiliations:** a Niels Bohr Institute, University of Copenhagen, Universitetsparken 5, 2100 Copenhagen E, Denmark; b Department of Plant and Environmental Sciences, University of Copenhagen, 1871 Frederiksberg C, Denmark

**Keywords:** small-angle scattering, size-exclusion chromatography, phospholipid nanodiscs, model refinement

## Abstract

The combination of online size-exclusion chromatography and small-angle X-ray scattering (SEC–SAXS) is rapidly becoming a key technique for structural investigations of elaborate biophysical samples in solution. Here, a novel model-refinement strategy centred around the technique is outlined and its utility is demonstrated by analysing data series from several SEC–SAXS experiments on phospholipid bilayer nanodiscs. Using this method, a single model was globally refined against many frames from the same data series, thereby capturing the frame-to-frame tendencies of the irradiated sample. These are compared with models refined in the traditional manner, in which refinement is based on the average profile of a set of consecutive frames from the same data series without an in-depth comparison of individual frames. This is considered to be an attractive model-refinement scheme as it considerably lowers the total number of parameters refined from the data series, produces tendencies that are automatically consistent between frames, and utilizes a considerably larger portion of the recorded data than is often performed in such experiments. Additionally, a method is outlined for correcting a measured UV absorption signal by accounting for potential peak broadening by the experimental setup.

## Abstract 2

A framework is presented for refining models from several data frames from the same size-exclusion chromatography small-angle scattering experiment. The method can be employed to drastically reduce the number of parameters refined from the data series.

## 1. Introduction

Small-angle scattering (SAS) is a well established and widely applied method that is used to investigate a broad range of soluble samples, ranging from particles of biomolecular origin, including proteins and nucleotide-based structures, to self-assembled systems such as micelles, vesicles and various lipid–protein complexes, including nanodiscs. The use of small-angle scattering for investigating biomolecular structures has triggered large improvements on both the instrument and the sample-environment sides. These improvements have been driven by the frequent scarcity of sample and the relatively small signal over the background, as well as the propensity of many biomolecular samples to aggregate.

The combination of size-exclusion chromatography (SEC) and small-angle X-ray scattering (SAXS) into an integrated SEC–SAXS setup and, more recently, of SEC and small-angle neutron scattering (SANS) into SEC–SANS, are great examples of such improvements (David & Pérez, 2009 ▸; Mathew *et al.*, 2004 ▸; Watanabe & Inoko, 2009 ▸; Jordan *et al.*, 2016 ▸; Johansen *et al.*, 2018 ▸). Despite the fact that SEC–SAS leads to a dilution of the sample and hence a decreased signal over the background, this is in most cases counterbalanced as the remaining part of the signal comes from a single species or a narrow distribution of species, making the data interpretation less ambiguous.

With the introduction of SEC–SAXS and SEC–SANS, size-exclusion-based segregation splits the sample into size-sorted fractions from which data are then continuously recorded by SAXS or SANS. Using this setup on a polydisperse sample, the investigator will obtain much more information than if the SAS analysis is performed on the nonfractionated sample. For example, for pure protein samples which are prone to oligomerization this setup may be used to separate and collect information on the different oligomeric states of the protein (Pedersen *et al.*, 2021 ▸). Usually, SEC–SAXS and SEC–SANS are used with the goal of overcoming protein-aggregation issues since the sample is irradiated immediately after SEC purification (Johansen *et al.*, 2018 ▸; Jeffries *et al.*, 2016 ▸; Ryan *et al.*, 2018 ▸). In these cases there is a narrow focus on a single species.

There are circumstances in which SEC fails to fully separate molecules with differing structures. Initial SEC–SAS data processing often involves checking for monodispersity within the relevant peak in the chromatogram by calculating radii of gyration (*R*_*g*_) or molecular weight (MW) per frame. The use of a program such as *CHROMIXS* (Panjkovich & Svergun, 2018 ▸), for example, makes this process very simple. Using this information, typically the average of a small set of consecutive frames are selected for further analysis. Usually the rest of the SEC–SAS data series is not analysed in depth, despite possibly also containing relevant information about the species. Furthermore, in cases where two or more discrete populations are merged into a single chromatographic peak there are advanced mathematical techniques available, such as state-of-the-art evolving factor analysis (EFA) software (Hopkins *et al.*, 2017 ▸; Konarev *et al.*, 2022 ▸; Tully *et al.*, 2021 ▸), to devolve the overlapping peaks and isolate SAXS profiles corresponding with each population. This is less applicable, however, to the naturally occurring polydispersity around a single species in self-assembled systems.

**Figure 1:**
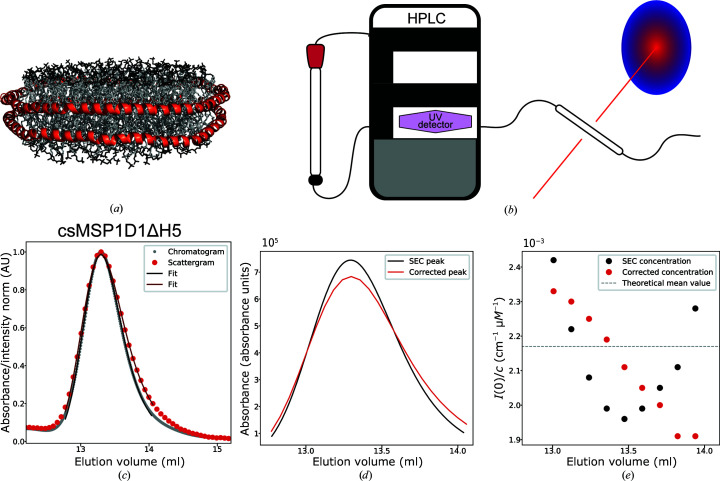
The experimental setup and broadening of the peak during SEC–SAXS. (*a*) Molecular visualization of a DMPC-loaded csMSP1D1ΔH5 nanodisc built with CHARMM-GUI *NanodiscBuilder* (Jo *et al.*, 2008 ▸; Qi *et al.*, 2019 ▸). (*b*) Schematic of the SEC–SAXS setup to reiterate the distance between the HPLC UV280 absorbance detector and the capillary where SAXS is recorded. (*c*) Normalized chromatogram and scattergram for csMSP1D1ΔH5 nanodiscs. The grey points indicate UV absorbance and the red points indicate the total intensity per frame. Solid lines are exponentially modified Gaussian (EMG) fits to the data. The centres of the two peaks are aligned. (*d*) The black profile is the EMG fit to the chromatogram in absorbance units. The red profile is the corrected version substituting in parameters from the fit to the scattergram while keeping the area under the curve constant. (*e*) *I*(0)/*c* as a function of the elution volume. Black points are calculated from the original SEC profile. Red points are calculated from the corrected profile. The dashed line is the theoretical value estimated for 120 DMPC per nanodisc.

Nanodiscs are disc-shaped particles consisting of a central lipid bilayer encircled by two amphipathic membrane-scaffolding proteins (MSPs), as depicted in Fig. 1 ▸(*a*) (Bayburt *et al.*, 2002 ▸; Denisov *et al.*, 2004 ▸). Nanodiscs are formed by a self-assembly process involving detergent-solubilized lipids and MSPs. The self-assembly is initiated by removal of the detergent, making the lipids and MSPs form particles in a process that is highly dependent on the MSP and lipids of choice. In addition, membrane proteins can be included in the self-assembly, resulting in membrane protein-loaded nanodiscs. Due to the presence of lipids, nanodiscs are commonly used as a platform to study the structure and function of membrane proteins in a native-like environment (Denisov & Sligar, 2017 ▸).

In this article, we investigate and discuss how the large amount of information obtained in a SEC–SAXS experiment can be brought into play through global analysis of the data. We use dimyristoylphosphatidylcholine (DMPC)-loaded nanodiscs of three various sizes, facilitated through three next-generation circularized (Nasr *et al.*, 2017 ▸) and supercharged (Johansen *et al.*, 2019 ▸) membrane-scaffold proteins (csMSPs). Circularization refers to the covalent linkage of the MSP N- and C-termini in order to improve size homogeneity, while increasing the number of negatively charged residues enhances the solubility of the nanodisc. The smallest nanodisc that we investigate, csMSP1D1ΔH5, is approximately 8 nm in diameter (Hagn *et al.*, 2013 ▸), followed by csMSP1D1, which is approximately 10 nm in diameter (Hagn *et al.*, 2013 ▸), and finally csMSP1E3D1, which is 13 nm in diameter (Johansen *et al.*, 2019 ▸). The solution structures of these three nanodiscs have been studied previously by offline SEC purification and standard robot SAXS measurements (Johansen *et al.*, 2019 ▸, 2021 ▸), however, without a focus on the underlying size and shape distributions within the populations. In this study, we demonstrate that this kind of information is easily accessible via SEC–SAXS. To the obtained data we fit a simple geometrical model for the nanodiscs that we have used several times before (Skar-Gislinge & Arleth, 2011 ▸; Skar-Gislinge *et al.*, 2010 ▸, 2018 ▸).

Global fitting of multiple data sets is already used to investigate simultaneously acquired SAXS and SANS data through the fitting of a common model which is then calculated in the relevant contrast. This has been widely exploited and several examples are available in the literature for various types of systems, *i.e.* microemulsions (Arleth & Pedersen, 2001 ▸), nanodiscs (Skar-Gislinge *et al.*, 2010 ▸), the self-assembly of polymers into toroids (Hollamby *et al.*, 2016 ▸) and micelles (Mineart *et al.*, 2019 ▸), and in the case of specifically deuterated proteins in solution (Whitten *et al.*, 2007 ▸; Heller *et al.*, 2003 ▸).

A global fitting approach can also be used to analyse a series of data on the same sample where a subset of the model parameters are conserved throughout the series and others vary. For such shared parameters, a single value is refined for all data sets. For parameters which are not shared, a distinct value is refined for each data set. Such approaches have been applied to diverse cases of analysis of SAXS data from time-dependent fibrillating samples (Herranz-Trillo *et al.*, 2017 ▸; Ortore *et al.*, 2011 ▸), the variation of monomer–dimer equilibria with concentration (Blobel *et al.*, 2009 ▸), temperature-induced aggregation (Mariani *et al.*, 2010 ▸; Gonnelli *et al.*, 2020 ▸), a SANS analysis of the growth behaviour of SDS micelles (Arleth *et al.*, 2002 ▸) and even the analysis of both a series of SAXS data and a series of SANS data simultaneously (Sinibaldi *et al.*, 2008 ▸).

The global approach to model fitting has strength in that it ensures a more self-consistent analysis across data sets and with fewer parameters. Additionally, a larger amount of the acquired data are used to evaluate the proposed model and to determine the model parameters. The weakness lies in the added complexity of the modelling setup.

Overall, we show how the global fitting approach provides a more robust analysis of the obtained SEC–SAXS data for nanodiscs. As a part of this, we are able to rationalize the degree of lipid loading in the nanodiscs over the SEC peak. For the small csMSP1D1ΔH5 discs we find that there is very minimal size separation over the peak, but for the slightly larger csMSP1D1 discs as well as the even larger csMSP1E3D1 discs we observe how the SEC splits the sample up into discs with progressively higher to lower lipid-to-MSP stoichiometries. The geometric parameters of the nanodiscs over the SEC peak can be described with a linear frame-to-frame relationship in order to reduce the number of free parameters while still providing a detailed structural overview of the nanodisc populations and without compromising the integrity of the fit to the data sets. The global model provides excellent fits to the whole series of eight SAXS data sets from the same SEC peak simultaneously for each of our three samples. Using our global model we are able to reduce the number of free parameters to 16, compared with 56 free parameters if we were to refine the nanodisc model against eight SAXS frames independently.

As a side note, we introduce a novel approach for quantifying the broadening of the peak during a SEC–SAXS experiment, with the aim of calculating more accurate concentration estimates, which are essential for modelling on an absolute scale.

## 2. Materials and methods

### 2.1. Sample preparation

MSP-based nanodiscs were prepared as described elsewhere (Johansen *et al.*, 2021 ▸), excluding the final size-exclusion chromatography (SEC) purification. Briefly, DMPC was solubilized to 50 m*M* with reconstitution buffer (20 m*M* Tris–HCl pH 7.5, 150 m*M* NaCl) containing 100 m*M* sodium cholate. The solubilized DMPC was mixed with MSP in molar ratios of 55:1 (csMSP1D1ΔH5), 80:1 (csMSP1D1) and 130:1 (csMSP1E3D1) and was diluted with reconstitution buffer to a final DMPC concentration of 10 m*M*. The samples were incubated at 28°C with 15%(*w*/*v*) detergent-absorbing beads (Amberlite XAD-2, Merck) for three hours. The samples were separated from the beads, stored on ice and transported to the SAXS facility.

### 2.2. Data acquisition

SAXS data were collected on BM29 at the European Synchrotron Radiation Facility (ESRF) using the online SEC–SAXS setup (Pernot *et al.*, 2013 ▸), where the temperature of the SAXS capillary was kept at 10°C. 200 µl samples were loaded onto a Superdex 200 Increase 10/300 GL column (GE) equilibrated in phosphate buffer. For csMSP1D1ΔH5 and csMSP1E3D1 nanodiscs the buffer was 20 m*M* sodium phosphate pH 7.0, 150 m*M* NaCl, while for csMSP1D1 nanodiscs the buffer was phosphate-buffered saline (Sigma) with 1 m*M* DTT. We note that nanodiscs were initially reconstituted in Tris-based buffer according to standardized procedures; however, the p*K*_a_ of Tris is quite temperature-sensitive, and to keep the pH stable in our measurements we opted for buffer exchange into phosphate buffer, which is rather insensitive to temperature. 1 s SAXS frames were continuously measured during sample elution. The intensity was measured as a function of *q*, with *q* = 4πsinθ/λ, where θ is half the scattering angle and λ is the wavelength (here 0.9919 Å), and calibrated to units of cm^−1^ using H_2_O as a calibration standard (Orthaber *et al.*, 2000 ▸). The absorbance at 280 nm was converted to a concentration using protein extinction coefficients calculated with *ProtParam* (Gasteiger *et al.*, 2005 ▸): 18 450 *M* ^−1^ cm^−1^ for csMSP1D1ΔH5 and csMSP1D1 and 26 930 *M* ^−1^ cm^−1^ for csMSP1E3D1. Note that DMPC does not absorb light at this wavelength. The loading nanodisc concentrations were 0.06 m*M* for csMSP1D1 and csMSP1E3D1 nanodiscs and 0.16 m*M* for csMSP1D1Δh5 nanodiscs.

### 2.3. Data processing

To reduce the size of the data series, the 1 s SAXS frames were averaged over 10 s. 50 frames collected prior to the elution peak, corresponding to buffer, were then averaged and used for background subtraction. The baseline intensity remains stable before and after the peak, indicating that the chosen buffer frames are suitable for the entire data series (see, for example, Supplementary Fig. S1). SAXS data were rebinned to lie evenly on a logarithmic *q*-scale. Pair-distance [*p*(*r*)] distributions were obtained by the indirect Fourier transform (IFT) method using the online program *BayesApp* available at https://genapp.rocks/ (Savelyev & Brookes, 2019 ▸; Hansen, 2000 ▸). Radii of gyration (*R*_*g*_) and the forward scattering [*I*(0)] were calculated using *AUTORG* from *ATSAS* (Petoukhov *et al.*, 2007 ▸). Scattergrams were generated by calculating the total intensity in the *q*-range 0.008–0.3 Å^−1^ per SAXS frame and plotting it as a function of elution volume, where we use the HPLC flow rate to convert SAXS time stamps to elution volumes so that the scattergrams and chromatograms can be aligned. The nanodisc model is implemented in *WillItFit* (Pedersen *et al.*, 2013 ▸).

### 2.4. Small-angle scattering and principles of the modelling

#### 2.4.1. Modelling of nanodiscs

**Figure 2:**
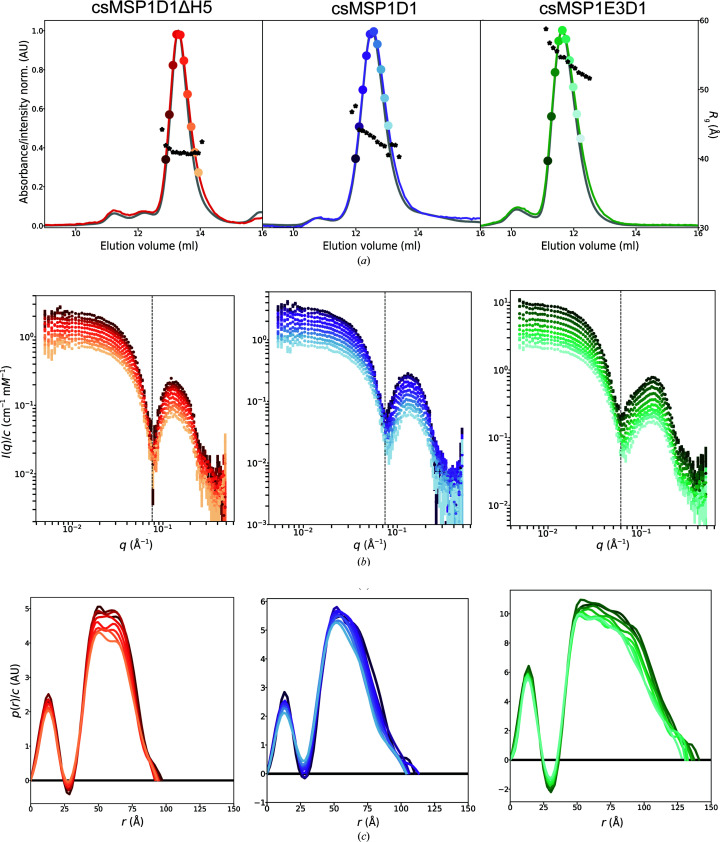
SEC–SAXS data indicating structural changes across the size-separated nanodisc samples. (*a*) Normalized SEC elution profiles scaled with SAXS scattergrams. The grey lines indicate UV absorbance at 280 nm. Solid coloured lines indicate the total intensity per frame. The black stars indicate the *R*_g_ per frame. (*b*) Series of scattering profiles from various positions in the SEC peak, normalized by concentration, where colours correspond to the highlighted frames in (*a*). The topmost data sets are on an absolute scale, while those below are scaled by 1.1^−*n*^, where *n* is the frame number. The black dashed line indicates the position of the first minimum of the top scattering profile. (*c*) *p*(*r*) distributions corresponding to the highlighted frames, normalized by concentration.

**Figure 3:**
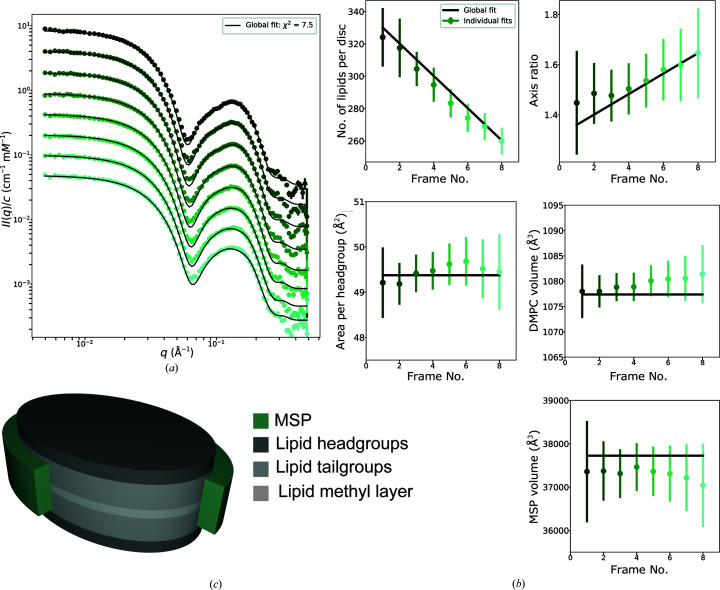
Model fit results for csMSP1E3D1 nanodiscs. (*a*) Global fit to experimental SAXS data sets from frames with increasing elution volumes/positions across the SEC peak. Data sets are the middle eight highlighted frames in Fig 2 ▸(*a*). (Individual fits are shown in Supplementary Fig. S3.) The topmost data set is on an absolute scale, while those below are scaled by 2^−*n*^, where *n* is the frame number. (*b*) Refined structural parameters. The coloured data points indicate parameters refined from each data set individually. The black lines indicate parameters refined from the global fit, where one shared value is found for *A*_L_, *v*_P_ and *v*_L_, while *N*_L_ and ɛ are both forced to follow a linear trend. (*c*) Representation of the nanodisc model used; a quarter of the MSP belt is not shown to highlight the interior structure of the lipid bilayer.

With our SAS data, our main objective is to refine structural models of our nanodiscs from the SEC–SAXS data presented in Fig. 2 ▸. The model of choice in this study is the well established nanodisc model (Skar-Gislinge *et al.*, 2010 ▸; Skar-Gislinge & Arleth, 2011 ▸), in which the geometric structure of the nanodisc is described by a series of form-factor amplitudes, each of which accounts for the scattering from a distinct part of the nanodisc. The nanodisc model is sketched in Fig. 3 ▸(*c*). These form-factor amplitudes have been mathematically described in the literature (Pedersen, 1997 ▸). The model is calculated on an absolute scale by utilizing the sample concentration, as well as the molecular composition of the MSP and DMPC, to calculate the scattering length applicable for each part of the nanodisc, as listed in Supplementary Table S7.

Overall, the nanodisc model is described by the following quantities: (i) the axis ratio of the patch of lipid bilayer, ɛ, (ii) the average area per phospholipid headgroup in the bilayer, *A*_L_, (iii) the number of lipids in a nanodisc, *N*_L_, (iv) the partial specific molecular volume of a phospholipid, ν_L_, (v) the partial specific molecular volume of an MSP, ν_P_, and (vi) the height of the cylinder describing the protein belt. In this study, we fix this height at 25.8 Å throughout our refinement, in line with previous studies (Bibow *et al.*, 2017 ▸). The model is sketched in Fig. 3 ▸. Additionally, we refine a constant background contribution, *b*, and a term accounting for the interface roughness in our model, *R* (Als-Nielsen & McMorrow, 2011 ▸). We denote this set of parameters as **θ**.

Such models are usually refined by minimizing the (reduced) 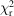 , which estimates the overlap between the data and a specified model function, *I*_Mod_(*q*, **θ**). This quantity is defined as

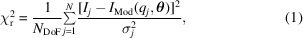

where *q*_*j*_, *I*_*j*_ and σ_*j*_ constitute the *j*th data point in a data set consisting of *N* data points. *N*_DoF_ is the number of degrees of freedom, which we compute as the number of data points minus the number of parameters in the model.

#### 2.4.2. Global fitting of multiple frames

In this study, we refined our structural models from several data sets simultaneously and found the best fit for the whole series. As our data sets were collected across a peak in the same SEC experiment, we split our list of parameters into two categories: parameters that we assumed to vary across the irradiated SEC fractions and parameters that we assumed not to vary. All nanodiscs within the same sample comprise the same lipids and MSPs, and hence there should be minimal variation in the volumes of lipids and MSPs. Although there is evidence to suggest that the dynamics and packing of lipids embedded in nanodiscs vary depending on the distance of the lipid from the rim (Bengtsen *et al.*, 2020 ▸; Martinez *et al.*, 2017 ▸), on average the area per headgroup should remain stable under identical experimental conditions. Rather, depending on sample preparation, there may be a distribution of fully loaded circular discs and under-loaded elliptical discs (Skar-Gislinge *et al.*, 2018 ▸). Thus for the *k*th data set we refine individual values of *N*_L_, ɛ and *b* (which we denote by **θ**_*k*_). The parameters ν_L_, ν_P_, *A*_L_ and *R* are refined to a single value used in all of the models; we label these parameters **Θ**.

In order to accommodate for this categorization of our parameters, we redefine our figure of merit, 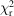 , from equation (1) to 

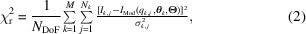

where *N*_*k*_ is the number of data points in the *k*th data set, of which there are *M*, which now prompts us to denote the *j*th data point in the *k*th data set by (*q*_*k*,*j*_, *I*_*k*,*j*_, σ_*k*,*j*_). Note that the model function now depends on not only the parameters specific to the *k*th data set, **θ**_*k*_, but also the ‘global’ parameters that are identical across all of the data sets, **Θ**. This is an adaptation of a similar scheme to analyze temperature series of SAXS data (Johansen *et al.*, 2021 ▸).

Additionally, rather than allowing the individual parameters in **θ**_*k*_ to vary irrespective to the other data sets, this scheme allows us to assume and enforce, for example, linear trends between the various frames to lower the total number of parameters refined in the scheme: *i.e.* rather than refining *M* individual values of *N*_L_, we assume a linear trend across the SEC fractions, *N*_L_ = *an* + *b*, where *n* is the frame number in the data series and *a* and *b* are parameters to be refined. Hence, we reduce the number of parameters in the refinement scheme by *M* − 2. By employing the same idea for ɛ, we reduce the number of refined parameters by an additional *M* − 2. In a sense, this notion is a natural extension of the idea of the ‘global’ parameters in **Θ** which are simply kept constant across the frames, and hence their frame-to-frame relationship is described by a single parameter using a zeroth-order polynomial rather than two parameters in a first-order polynomial. We remark that a linear function is sufficient for our purposes; providing a more physical model to describe particles eluting from a SEC column could require a more complicated relationship and further investigation is necessary before drawing conclusions. More complicated relationships can readily be employed but become impractical (or simply useless) if they require a number of coefficients comparable to the number of data sets, unless there is a solid underlying theory to support their use.

## 3. Results and discussion

### 3.1. Co-calibration of the SEC–UV280 and the SEC–SAXS intensities

The SEC–SAXS setup is sketched in Fig. 1 ▸(*b*). Broadening of the elution peak often occurs during SEC–SAXS experiments due to Taylor dispersion (Taylor, 1953 ▸) and the difference in diameter between the HPLC tubing and the SAXS capillary (Bucciarelli *et al.*, 2018 ▸). Here, we introduce a novel approach for estimating and correcting for this broadening. The approach is illustrated in Figs. 1 ▸(*c*) and 1 ▸(*d*) and Supplementary Fig. S2. In Fig. 1 ▸(*c*) the normalized chromatogram for csMSP1D1ΔH5 nanodiscs is plotted with its corresponding scattergram, *i.e.* the scattering intensity per individual frame as a function of the elution volume. The centre of the peak of the scattergram is aligned with the centre of the peak of the chromatogram and the broadening of the scattergram is clearly visible.

Exponentially modified Gaussian (EMG) functions are good models for chromatographic peaks under a range of conditions (Naish & Hartwell, 1988 ▸; Busnel *et al.*, 2001 ▸), where broadening can be characterized by two parameters: the standard deviation (width), σ, and a relaxation parameter (skew), τ. EMGs were fitted to the main peaks of the chromatogram and scattergram via nonlinear least-squares regression. A ‘corrected’ SEC profile was then calculated by keeping the area under the EMG fit of the chromatogram constant, but substituting in σ and τ from the fit to the scattergram in order to take account of the change in the shape of the peak, which becomes wider and develops a tail on the right-hand side. Thus, the corrected profile approximates the UV absorption as if it were recorded directly on the SAXS capillary and should provide much more accurate concentration estimates. The original SEC peak and the corrected SEC peak can be compared in Fig. 1 ▸(*d*). Estimating the sample concentration directly from the raw HPLC absorption measurements may lead to underestimated concentrations in the tails of the peak and overestimated concentrations in the centre.

As a check, we calculated the forward scattering *I*(0) divided by concentration for the SAXS data sets as a function of elution volume, as plotted in Fig. 1 ▸(*e*). The values calculated with the original concentrations show a prominent decrease and then an increase, which cannot be readily explained. For a fully homogeneous sample, *I*(0)/*c* should remain constant. If there is some size variation *I*(0)/*c* may decrease systematically towards the right-hand side, which is seen for the values calculated with the corrected concentrations. These values also fall close to an estimate of *I*(0)/*c* which we calculated for csMSP1D1ΔH5 nanodiscs loaded with 120 DMPC. We note that during modelling the nanodisc form factor multiplied by the new concentrations matches the experimental SAXS intensities perfectly without the need for an additional scaling factor.

One potential drawback of this method lies in the fact that scattering intensity scales with squared particle volume while protein UV absorption does not, meaning that some discrepancy between the shapes of the chromatograms and the scattergrams is to be expected. In this case, however, the corrected SEC profile performed better and the method could be considered for other SEC–SAXS studies in which accurate concentration estimates are desirable for absolute-scale modelling or molecular-weight determination.

### 3.2. SEC–SAXS data overview

The SEC–SAXS data and associated *p*(*r*) distributions for all three nanodiscs species are shown in Fig. 2 ▸. For each nanodisc species the data indicate some systematic structural variation across the size-sorted fractions. For the smallest nanodiscs, *R*_g_ stays constant across the SEC peak at ∼40 Å; however, for csMSP1D1 nanodiscs there is a steady decrease from ∼46 to 42 Å, and for csMSP1E3D1 nanodiscs the decrease from ∼58 to 52 Å is even more apparent. Each of the scattering curves is compatible with that we typically observe for monodisperse nanodiscs: a flat Guinier region in the low-*q* regime, followed by a trough and a broad bump at medium to high *q*. csMSP1D1ΔH5 and csMSP1E3D1 display the typical nanodisc double-bump feature (Skar-Gislinge *et al.*, 2010 ▸; Denisov *et al.*, 2005 ▸). For csMSP1D1, and even more significantly for csMSP1E3D1, as the position of the fraction in the elution profile progresses, the first minimum in the scattering curve shifts systematically to higher *q* values, indicating a change in particle shape. The *p*(*r*) distributions reaffirm this, showing a systematic loss of depth of the first minimum alongside a decrease in the maximum pair distance (*D*_max_) as we move to larger elution volumes. Again, these variations are least prominent in the small discs and most prominent in the large discs, which may suggest that larger discs are more structurally disperse. Altogether, these observations suggest that even within a SEC-purified nanodisc population there is some size distribution which may be sorted by a SEC column so that larger particles elute first, but below some resolution it will not be separated into multiple elution peaks.

### 3.3. Modelling and data analysis

Analysing many data sets from the same SEC–SAXS experiment with the nanodisc model provides more detailed insights into the size and shape distributions underlying the populations. We select eight sequential SAXS data sets for each sample. Firstly, we refine the model against each data set independently as a precursor. Secondly, we refine the model against each data set *simultaneously* with both global and frame-specific free parameters in order to constrain the fits further and investigate the amount of information which can be extracted with a reduced number of free parameters. We note that although each of the individual data sets are collected from a narrow fraction of the SEC-purified sample, the data sets may still contain some slight overlap between different nanodisc sizes. The refined model therefore describes the average scattering from the nanodiscs present and does not account for polydispersity within a certain frame.

#### 3.3.1. Individual fits

When fitted to the individual frames, the nanodisc model provides excellent fits to each of the SAXS data sets chosen for further analysis. The individual fits are plotted in Supplementary Figs. S3, S4 and S5. The refined model parameters from individual fits to the eight SAXS data sets for csMSP1E3D1 nanodiscs are plotted as coloured points in Fig. 3 ▸(*b*) and are further listed in Supplementary Table S1. The results for csMSP1D1 and csMSP1D1ΔH5 nanodiscs are given in the supporting information.

For all three nanodisc samples the area per lipid headgroup, *A*_L_, and the partial specific molecular volumes of the lipid, *v*_L_, and MSP, *v*_P_, generally fluctuate only slightly between frames. This is in line with our expectations since the volume of DMPC and of each MSP should be very stable for the entire sample, regardless of elution volume. Although prone to local fluctuations, the refined value of the area per headgroup should also remain stable. For the three nanodiscs, *A*_L_ was refined to values of between 49.5 and 53.5 Å^2^, which is in good agreement with previous values of 47.5 Å^2^ for DMPC-loaded nanodiscs (Johansen *et al.*, 2021 ▸), 52.1 Å^2^ for DMPC-loaded peptide discs (Midtgaard *et al.*, 2014 ▸) and 47.2 Å^2^ for a pure DMPC bilayer (Tristram-Nagle *et al.*, 2002 ▸), all of which were recorded at 10°C. We mention that since the temperature is not controlled over the entire SEC–SAXS instrumentation, the temperature of the sample may be slightly above 10°C. This may affect the lipid packing slightly; however, as the temperature was kept below the melting temperature of DMPC at 24°C the effect will not be prominent (Johansen *et al.*, 2021 ▸). *v*_L_ becomes up to 5% larger than the reported value of 1041 Å^3^ (Tristram-Nagle *et al.*, 2002 ▸). *v*_P_ stays within 5% below our pre-estimated values based on the molecular compositions, which are specific for each MSP. Prominent frame-to-frame fluctuations of these three free parameters could be the result of overfitting to the SAXS data and strong correlations between parameters in the model.

Rather, the systematic variations in the SAXS data sets are reflected in the steady decrease in the number of lipids per nanodisc, *N*_L_, as a function of elution volume, likely coinciding with a general increase in the axis ratio, ɛ. Since the circumference of the nanodisc is determined by the length of the MSP and is therefore expected to remain constant, variation in the number of lipids (and thereby the bilayer surface area) must be compensated by some variation in the shape of the disc. Although ɛ is poorly determined by this method, we assume that this dependency between *N*_L_ and ɛ is present across the sample. Each data set indicates elliptical discs, where discs with higher lipid-to-MSP stoichiometries appear to be slightly rounder, while discs with lower lipid-to-MSP stoichiometries become more elliptical. The same trend has been observed many times (Skar-Gislinge *et al.*, 2010 ▸, 2018 ▸; Graziano *et al.*, 2018 ▸). According to our analysis, csMSP1E3D1 nanodiscs contain the largest underlying size distribution, with a difference in *N*_L_ of 65 lipids between the size-sorted first and last frames, from 325 to 260 lipids. csMSP1D1 decreases by 35 lipids from 150 to 115 and csMSP1D1ΔH5 decreases by 15 lipids from 130 to 115.

Unlike previous reports (Johansen *et al.*, 2021 ▸), we do not see a simple linear correlation between axis ratio and length of the MSP here, despite larger discs theoretically being more structurally flexible. csMSP1D1 nanodiscs persistently have the largest axis ratio, which varies between 1.6 and 1.8, whereas csMSP1D1ΔH5 nanodiscs have the smallest, varying between 1.3 and 1.5, and csMSP1E3D1 lies in between with values varying between 1.45 and 1.65. Although seemingly incidental, this coincides with a recent course-grained molecular-dynamics study of the same circularized MSPs (cMSPs, non­supercharged; Kjølbye *et al.*, 2021 ▸), where cMSP1D1 was found to have the highest degree of anisotropy, with cMSP1D1ΔH5 being the most circular and cMSP1E3D1 falling in between. These results suggest that there are other factors influencing the shape of nanodiscs besides the degree of lipid loading, especially the choice of MSP and its intrinsic rigidity.

#### 3.3.2. Global fits

Fitting the nanodisc model to *M* data sets requires 7*M* free parameters. Certain parameters, however, should be conserved when examining data sets from the same SEC–SAXS experiment and hence fitting the parameter *M* times becomes redundant. The individual fits justify the introduction of global parameters for *A*_L_, *v*_L_, *v*_P_ and *R* to ensure that the model refinement is self-consistent and that these parameters are better determined. *N*_L_ and ɛ, however, capture important trends between the data sets as a function of elution volume. This information would be lost if fitting using a constant rather than the two-parameter function that we utilized here.

A global model *could* be set up with *A*_L_, *v*_L_, *v*_P_ and *R* as global parameters and *N*_L_, ɛ and *b* as frame-specific parameters, such that the number of free parameters is 4 + 3*M*. However, to constrain the fit even further, frame-to-frame linear relationships are enforced for *N*_L_ and ɛ, where the *y* intercept and slope of the respective functions are global parameters as described in Section 2.4.2 and shown in the top row in Fig 3 ▸(*b*), capturing increasing or decreasing trends across the data series using only two free parameters per function. In this implementation of the model, the number of free parameters is 8 + *M*, where the only frame-specific parameter is the background, *b*. In this case, where *M* = 8, swapping from individual modelling to the global modelling described here drastically reduces the number of free parameters from 56 (7 × 8) to 16 (8 + 8).

Fig. 3 ▸(*a*) shows the global fit refined against the eight SAXS data sets simultaneously for csMSP1E3D1 nanodiscs. The refined model parameters are listed in Supplementary Table S1 and the frame-to-frame relationships are plotted in Fig. 3 ▸(*b*) as solid black lines. Global results for csMSP1D1 and csMSP1D1ΔH5 nanodiscs are given in the supporting information. Despite the extra constraints, the global model is able to describe the entire series of SAXS data sets excellently, with no features standing out visually as poorly captured. The global model achieves impressive 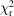 values of 7.5, 5.4 and 5.9 for csMSP1E3D1, csMSP1D1 and csMSP1D1ΔH5, respectively, as calculated by equation (2). Furthermore, reasonable structural parameters are maintained over the three samples and the important frame-to-frame trends are sustained.

For csMSP1E3D1 and csMSP1D1ΔH5 the global fit parameters mimic the individual fit parameters very closely, which suggests that the results are reliable and the choice of frame-specific and global parameters are compatible. For csMSP1D1 the global fit parameters, although still satisfactory, are a slightly looser match to the individual fit parameters, especially the axis ratio, where the global model possibly determines a much steeper slope. We note that this could be explained by the fact that this data series has the poorest signal-to-noise ratio. We further comment that the large error on the ɛ slope for all three experiments should be expected since it is clear in the individual fits that ɛ is poorly determined and a range of slopes could be applicable. Refined global fit parameters should not be anticipated to emerge as the exact mean of the individual fit results, since the global fit minimizes the risk of overfitting to the SAXS data and constrains correlations between fit parameters.

We observe that the refined gradient of the straight line representing the fraction-dependent change in the number of lipids, *N*_L_, further rationalizes the degree of polydispersity present in each respective nanodisc sample: the largest disc csMSP1E3D1 shows the greatest gradient of −9.96*N*_L_ per frame, with csMSP1D1 showing a gradient of −5.00*N*_L_ per frame and csMSP1D1ΔH5 showing the most gentle gradient of −1.47*N*_L_ per frame. These slopes can be compared with linear fits to *R*_g_ as a function of position, where we calculate slopes of −0.23, −0.18 and −0.03 Å per frame for csMSP1E3D1, csMSP1D1 and csMSP1D1ΔH5, respectively.

Summing up, employing frame-to-frame constraints in our analysis of the presented SEC–SAXS data seems to allow considerably more constrained fits of a large amount of data whilst still producing realistic models and capturing inter-frame trends in a quantitative manner. The most notable advantages are the considerable reduction in the total number of parameters refined from the data and the tractability of refining a single model accounting for all of the data sets rather than individual models from each data set, which are then to be compared at a later stage; both of which in the cases presented here seem to come at little expense in terms of the quality of the fits.

## 4. Conclusions and further perspectives

Often during SEC–SAXS analysis only a small fraction of the SEC peak is considered and a large amount of structural information is discarded. We perform a comprehensive investigation into three types of next-generation nanodiscs by analysing many SAXS data sets from the same SEC–SAXS experiment. The size-sorted SAXS data sets reveal some systematic polydispersity within the structure of the nanodisc populations. A global approach to model fitting provides a robust analysis to help characterize the polydispersity. We observe that the SEC column gradually splits the samples into discs with high and low lipid-to-MSP stoichiometries. We employ simple frame-to-frame linear functions to further reduce the number of free parameters in the fitting routine. Despite the extra constraints, the global model is able to describe the entire series of SAXS data sets excellently and provides a detailed overview of the nanodisc populations through frame-specific and global refined values.

The reduction in the number of parameters refined from the data sets is a particularly attractive attribute of the outlined modelling scheme. Like similar inference tasks, model refinement from small-angle scattering data is prone to overfitting, so these simplifications (in terms of number of parameters in the model) provide a convenient means of analyzing the extensive amount of data one obtains from, for example, a SEC–SAXS experiment in a somewhat constrained manner. Naturally, such schemes rely intrinsically on the validity of the assumed trends across the analyzed data sets. Here, we successfully employ constant and linear relationships and argue that they are indeed sufficient to capture the general behavior of our data; mostly as we observe little to no increase in our figure of merit and the overall quality of our fits by employing them.

Our method has general applicability for samples and systems with inherent polydispersity within the resolution of the SEC column, including cases where the SEC peak is asymmetric or where two peaks have merged together. These include nanodiscs, as presented here, as well as similar membrane-protein carrier systems, including di-block co­polymer lipid particles, for example, styrene–maleic acid lipid particles (Knowles *et al.*, 2009 ▸), saposin lipid particles (Frauenfeld *et al.*, 2016 ▸) and detergent micelles. Additionally, the method could be modified to analyse biological systems in different types of equilibrium and where distinct populations cannot be sufficiently separated on SEC for individual analysis (Vestergaard, 2016 ▸). These include, for example, protein monomer–dimer equilibria, protein–ligand equilibria, phase-separated disordered proteins or systems adopting different structural states. In these cases, our method could be complementary to the popular evolving factor analysis (EFA) programs where model-independent EFA can be employed to identify and isolate uncontaminated profiles of the distinct populations for further structural analysis, potentially including global fitting (although of only two or three data sets). With EFA it is possible to extract an overall picture of sources of extreme structural heterogeniety within a sample. Previous examples include identifying scattering contributions from massive contaminants (Meisburger *et al.*, 2016 ▸), separating protein monomers from dimers or oligomers (Hopkins *et al.*, 2017 ▸; Konarev *et al.*, 2022 ▸) and separating bound and unbound protein states (Tully *et al.*, 2021 ▸). Our presented method is more suitable, however, when the desired outcome is a continuous description of systematic polydispersity across a data series, particularly when there is an underlying distribution within a single population or when the amount of polydispersity is too small for EFA to detect. This is only possible by investigating many narrow fractions of the elution profile. Furthermore, EFA fails when the chromatographic peak is too asymmetrical or when two peaks are too close together (Konarev *et al.*, 2022 ▸). In this work we analyse data sets directly from SEC–SAXS and assume that each fraction contains only a single population; however, one should be cautious since this is not necessarily true under the resolution of the SEC column.

Furthermore, our global fitting scheme is readily suitable for SEC–SANS experiments, and would be a very powerful fitting platform if the model could be refined against series of SEC–SAXS data sets and series of SEC–SANS data sets simultaneously. Finally, issues with peak broadening are well acknowledged in the SEC–SAXS community (Ryan *et al.*, 2018 ▸) and efforts have been made to measure the absorption directly on the SAXS capillary (Bucciarelli *et al.*, 2018 ▸). As part of our overall method, we suggest a simple correction procedure for the online absorption measurement, which eliminates parts of the problem with peak broadening and thereby allows more accurate determination of the forward scattering and thereby parameters such as molecular weight. 

## References

[bb1] Als-Nielsen, J. & McMorrow, D. (2011). *Elements of Modern X-ray Physics*, 2nd ed. Chichester: John Wiley & Sons.

[bb2] Arleth, L., Bergström, M. & Pedersen, J. S. (2002). *Langmuir*, **18**, 5343–5353.

[bb3] Arleth, L. & Pedersen, J. S. (2001). *Phys. Rev. E*, **63**, 061406. 10.1103/PhysRevE.63.061406 11415103

[bb4] Bayburt, T. H., Grinkova, Y. V. & Sligar, S. G. (2002). *Nano Lett.* **2**, 853–856.

[bb5] Bengtsen, T., Holm, V. L., Kjølbye, L. R., Midtgaard, S. R., Johansen, N. T., Tesei, G., Bottaro, S., Schiøtt, B., Arleth, L. & Lindorff-Larsen, K. (2020). *eLife*, **9**, e56518. 10.7554/eLife.56518 PMC7426092 32729831

[bb6] Bibow, S., Polyhach, Y., Eichmann, C., Chi, C. N., Kowal, J., Albiez, S., McLeod, R. A., Stahlberg, H., Jeschke, G., Güntert, P. & Riek, R. (2017). *Nat. Struct. Mol. Biol.* **24**, 187–193. 10.1038/nsmb.3345 28024148

[bb7] Blobel, J., Bernadó, P., Svergun, D. I., Tauler, R. & Pons, M. (2009). *J. Am. Chem. Soc.* **131**, 4378–4386. 10.1021/ja808490b 19275229

[bb8] Bucciarelli, S., Midtgaard, S. R., Pedersen, M. N., Skou, S., Arleth, L. & Vestergaard, B. (2018). *J. Appl. Cryst.* **51**, 1623–1632. 10.1107/S1600576718014462 PMC6276278 30546289

[bb9] Busnel, J. P., Foucault, F., Denis, L., Lee, W. & Chang, T. (2001). *J. Chromatogr. A*, **930**, 61–71. 10.1016/s0021-9673(01)01159-1 11681580

[bb10] David, G. & Pérez, J. (2009). *J. Appl. Cryst.* **42**, 892–900.

[bb11] Denisov, I. G., Grinkova, Y. V., Lazarides, A. A. & Sligar, S. G. (2004). *J. Am. Chem. Soc.* **126**, 3477–3487. 10.1021/ja0393574 15025475

[bb12] Denisov, I. G., McLean, M. A., Shaw, A. W., Grinkova, Y. V. & Sligar, S. G. (2005). *J. Phys. Chem. B*, **109**, 15580–15588. 10.1021/jp051385g PMC2518645 16852976

[bb13] Denisov, I. G. & Sligar, S. G. (2017). *Chem. Rev.* **117**, 4669–4713. 10.1021/acs.chemrev.6b00690 PMC5805400 28177242

[bb14] Frauenfeld, J., Löving, R., Armache, J.-P., Sonnen, A. F.-P., Guettou, F., Moberg, P., Zhu, L., Jegerschöld, C., Flayhan, A., Briggs, J. A. G., Garoff, H., Löw, C., Cheng, Y. & Nordlund, P. (2016). *Nat. Methods*, **13**, 345–351. 10.1038/nmeth.3801 PMC4894539 26950744

[bb15] Gasteiger, E., Hoogland, C., Gattiker, A., Wilkins, M., Appel, R. & Bairoch, A. (2005). *The Proteomics Protocols Handbook*, pp. 571–607. Totowa: Humana Press.

[bb16] Gonnelli, A., Pieraccini, S., Baldassarri, E. J., Funari, S., Masiero, S., Ortore, M. G. & Mariani, P. (2020). *Nanoscale*, **12**, 1022–1031. 10.1039/c9nr08556d 31845695

[bb17] Graziano, V., Miller, L. & Yang, L. (2018). *J. Appl. Cryst.* **51**, 157–166. 10.1107/S1600576717018441 PMC5822991 29507548

[bb18] Hagn, F., Etzkorn, M., Raschle, T. & Wagner, G. (2013). *J. Am. Chem. Soc.* **135**, 1919–1925. 10.1021/ja310901f PMC3566289 23294159

[bb19] Hansen, S. (2000). *J. Appl. Cryst.* **33**, 1415–1421.

[bb20] Heller, W. T., Finley, N. L., Dong, W.-J., Timmins, P., Cheung, H. C., Rosevear, P. R. & Trewhella, J. (2003). *Biochemistry*, **42**, 7790–7800. 10.1021/bi0341509 12820888

[bb21] Herranz-Trillo, F., Groenning, M., van Maarschalkerweerd, A., Tauler, R., Vestergaard, B. & Bernadó, P. (2017). *Structure*, **25**, 5–15. 10.1016/j.str.2016.10.013 27889205

[bb22] Hollamby, M. J., Aratsu, K., Pauw, B. R., Rogers, S. E., Smith, A. J., Yamauchi, M., Lin, X. & Yagai, S. (2016). *Angew. Chem.* **128**, 10044–10047. 10.1002/anie.201603370 27383466

[bb23] Hopkins, J. B., Gillilan, R. E. & Skou, S. (2017). *J. Appl. Cryst.* **50**, 1545–1553. 10.1107/S1600576717011438 PMC5627684 29021737

[bb24] Jeffries, C. M., Graewert, M. A., Blanchet, C. E., Langley, D. B., Whitten, A. E. & Svergun, D. I. (2016). *Nat. Protoc.* **11**, 2122–2153. 10.1038/nprot.2016.113 PMC5402874 27711050

[bb25] Jo, S., Kim, T., Iyer, V. G. & Im, W. (2008). *J. Comput. Chem.* **29**, 1859–1865. 10.1002/jcc.20945 18351591

[bb26] Johansen, N. T., Luchini, A., Tidemand, F. G., Orioli, S., Martel, A., Porcar, L., Arleth, L. & Pedersen, M. C. (2021). *Langmuir*, **37**, 6681–6690. 10.1021/acs.langmuir.1c00560 34038130

[bb27] Johansen, N. T., Pedersen, M. C., Porcar, L., Martel, A. & Arleth, L. (2018). *Acta Cryst.* D**74**, 1178–1191. 10.1107/S2059798318007180 30605132

[bb28] Johansen, N. T., Tidemand, F. G., Nguyen, T. T., Rand, K. D., Pedersen, M. C. & Arleth, L. (2019). *FEBS J.* **286**, 1734–1751. 10.1111/febs.14766 30675761

[bb29] Jordan, A., Jacques, M., Merrick, C., Devos, J., Forsyth, V. T., Porcar, L. & Martel, A. (2016). *J. Appl. Cryst.* **49**, 2015–2020. 10.1107/S1600576716016514 PMC5139991 27980509

[bb30] Kjølbye, L. R., De Maria, L., Wassenaar, T. A., Abdizadeh, H., Marrink, S. J., Ferkinghoff-Borg, J. & Schiøtt, B. (2021). *J. Chem. Inf. Model.* **61**, 2869–2883. 10.1021/acs.jcim.1c00157 34048229

[bb31] Knowles, T. J., Finka, R., Smith, C., Lin, Y.-P., Dafforn, T. & Overduin, M. (2009). *J. Am. Chem. Soc.* **131**, 7484–7485. 10.1021/ja810046q 19449872

[bb32] Konarev, P. V., Graewert, M. A., Jeffries, C. M., Fukuda, M., Cheremnykh, T. A., Volkov, V. V. & Svergun, D. I. (2022). *Protein Sci.* **31**, 269–282. 10.1002/pro.4237 PMC8740826 34767272

[bb33] Mariani, P., Spinozzi, F., Federiconi, F., Ortore, M. G., Amenitsch, H., Spindler, L. & Drevensek-Olenik, I. (2010). *J. Nucleic Acids*, **2010**, 472478. 10.4061/2010/472478 PMC2915817 20725625

[bb34] Martinez, D., Decossas, M., Kowal, J., Frey, L., Stahlberg, H., Dufourc, E. J., Riek, R., Habenstein, B., Bibow, S. & Loquet, A. (2017). *ChemPhysChem*, **18**, 2651–2657. 10.1002/cphc.201700450 PMC5697661 28573816

[bb35] Mathew, E., Mirza, A. & Menhart, N. (2004). *J. Synchrotron Rad.* **11**, 314–318. 10.1107/S0909049504014086 15211037

[bb36] Meisburger, S. P., Taylor, A. B., Khan, C. A., Zhang, S., Fitzpatrick, P. F. & Ando, N. (2016). *J. Am. Chem. Soc.* **138**, 6506–6516. 10.1021/jacs.6b01563 PMC4896396 27145334

[bb37] Midtgaard, S. R., Pedersen, M. C., Kirkensgaard, J. J. K., Sørensen, K. K., Mortensen, K., Jensen, K. & Arleth, L. (2014). *Soft Matter*, **10**, 738–752. 10.1039/c3sm51727f 24651399

[bb38] Mineart, K. P., Ryan, J. J., Appavou, M. S., Lee, B., Gradzielski, M. & Spontak, R. J. (2019). *Langmuir*, **35**, 1032–1039. 10.1021/acs.langmuir.8b03825 30609374

[bb39] Naish, P. J. & Hartwell, S. (1988). *Chromatographia*, **26**, 285–296.

[bb40] Nasr, M. L., Baptista, D., Strauss, M., Sun, Z. J., Grigoriu, S., Huser, S., Plückthun, A., Hagn, F., Walz, T., Hogle, J. M. & Wagner, G. (2017). *Nat. Methods*, **14**, 49–52. 10.1038/nmeth.4079 PMC5199620 27869813

[bb41] Orthaber, D., Bergmann, A. & Glatter, O. (2000). *J. Appl. Cryst.* **33**, 218–225.

[bb42] Ortore, M. G., Spinozzi, F., Vilasi, S., Sirangelo, I., Irace, G., Shukla, A., Narayanan, T., Sinibaldi, R. & Mariani, P. (2011). *Phys. Rev. E*, **84**, 061904. 10.1103/PhysRevE.84.061904 22304113

[bb43] Panjkovich, A. & Svergun, D. I. (2018). *Bioinformatics*, **34**, 1944–1946. 10.1093/bioinformatics/btx846 PMC5972624 29300836

[bb44] Pedersen, D. V., Pedersen, M. N., Mazarakis, S. M., Wang, Y., Lindorff-Larsen, K., Arleth, L. & Andersen, G. R. (2021). *eLife*, **10**, e63356. 10.7554/eLife.63356 PMC7857727 33480354

[bb45] Pedersen, J. S. (1997). *Adv. Colloid Interface Sci.* **70**, 171–210.

[bb46] Pedersen, M. C., Arleth, L. & Mortensen, K. (2013). *J. Appl. Cryst.* **46**, 1894–1898.

[bb47] Pernot, P., Round, A., Barrett, R., De Maria Antolinos, A., Gobbo, A., Gordon, E., Huet, J., Kieffer, J., Lentini, M., Mattenet, M., Morawe, C., Mueller-Dieckmann, C., Ohlsson, S., Schmid, W., Surr, J., Theveneau, P., Zerrad, L. & McSweeney, S. (2013). *J. Synchrotron Rad.* **20**, 660–664. 10.1107/S0909049513010431 PMC3943554 23765312

[bb48] Petoukhov, M. V., Konarev, P. V., Kikhney, A. G. & Svergun, D. I. (2007). *J. Appl. Cryst.* **40**, s223–s228.

[bb49] Qi, Y., Lee, J., Klauda, J. B. & Im, W. (2019). *J. Comput. Chem.* **40**, 893–899. 10.1002/jcc.25773 30677169

[bb50] Ryan, T. M., Trewhella, J., Murphy, J. M., Keown, J. R., Casey, L., Pearce, F. G., Goldstone, D. C., Chen, K., Luo, Z., Kobe, B., McDevitt, C. A., Watkin, S. A., Hawley, A. M., Mudie, S. T., Samardzic Boban, V. & Kirby, N. (2018). *J. Appl. Cryst.* **51**, 97–111.

[bb51] Savelyev, A. & Brookes, E. (2019). *Future Gener. Comput. Syst.* **94**, 929–936.

[bb52] Sinibaldi, R., Ortore, M. G., Spinozzi, F., de Souza Funari, S., Teixeira, J. & Mariani, P. (2008). *Eur. Biophys. J.* **37**, 673–681. 10.1007/s00249-008-0306-z 18365187

[bb53] Skar-Gislinge, N. & Arleth, L. (2011). *Phys. Chem. Chem. Phys.* **13**, 3161–3170. 10.1039/c0cp01074j 21152549

[bb54] Skar-Gislinge, N., Johansen, N. T., Høiberg-Nielsen, R. & Arleth, L. (2018). *Langmuir*, **34**, 12569–12582. 10.1021/acs.langmuir.8b01503 30239200

[bb55] Skar-Gislinge, N., Simonsen, J. B., Mortensen, K., Feidenhans’l, R., Sligar, S. G., Lindberg Møller, B., Bjørnholm, T. & Arleth, L. (2010). *J. Am. Chem. Soc.* **132**, 13713–13722. 10.1021/ja1030613 PMC4120756 20828154

[bb56] Taylor, G. (1953). *Proc. R. Soc. London Ser. A*, **219**, 186–203.

[bb57] Tristram-Nagle, S., Liu, Y., Legleiter, J. & Nagle, J. F. (2002). *Biophys. J.* **83**, 3324–3335. 10.1016/S0006-3495(02)75333-2 PMC1302408 12496100

[bb58] Tully, M., Tarbouriech, N., Rambo, R., Hutin, S., Tully, M. D. & Rambo, R. P. (2021). *J. Vis. Exp.*, e61578. 10.3791/61578 33586708

[bb59] Vestergaard, B. (2016). *Arch. Biochem. Biophys.* **602**, 69–79. 10.1016/j.abb.2016.02.029 26945933

[bb60] Watanabe, Y. & Inoko, Y. (2009). *J. Chromatogr. A*, **1216**, 7461–7465. 10.1016/j.chroma.2009.02.053 19269643

[bb61] Whitten, A. E., Jacques, D. A., Hammouda, B., Hanley, T., King, G. F., Guss, J. M., Trewhella, J. & Langley, D. B. (2007). *J. Mol. Biol.* **368**, 407–420. 10.1016/j.jmb.2007.01.064 17350039

